# Electric Transportation of Microbes

**Published:** 1913-11

**Authors:** 


Electric Transportation of Microbes. J. Comandon, *Arch. d'Elect. Med.,* July, 1913. Living microscopic objects, such as bacteria, blood corpuscles and protozoa, suspended in organic liquids, have an electric charge. By means of platinum plates set into slides, a galvanic current is passed through these suspensions, with resulting characteristic movements, which have been reproduced by micro-cinemetagraphic methods. Plates reproducing the cinemetograph pictures are shown, of the typhoid bacillus moving toward the cathode; of the trypanosomes of nagana in the blood of a mouse, moving toward the anode; of paramecies moving toward the cathode, etc. These movements depend upon the relations of the charges of the microbes and of the liquid of suspension and are quite characteristic, but not sufficiently so in all strains for diagnostic purposes. Cultures of the same species, made in the same manner, always show the same direction of movement under the galvanic current. The development of this method is exceedingly interesting and may prove to possess practical diagnostic value.

